# Translational Geroscience: Human Models of Healthy Aging and Longevity

**DOI:** 10.1093/geroni/igab046.1162

**Published:** 2021-12-17

**Authors:** Sofiya Milman

**Affiliations:** Albert Einstein College of Medicine, Bronx, New York, United States

## Abstract

While insulin like growth factor-1 (IGF-1) is a well-established modulator of aging and longevity in model organisms, its role in humans is less well understood. Previous ambiguities in part have been attributed to cohort characteristics and unawareness of interactions between age and IGF-1. Centenarians have emerged as an ideal model of healthy aging because they delay the onset of age-related diseases and often remain disease free for the duration of their lifespan. In cohorts of centenarians and generally healthy older adults, we demonstrated that reduced IGF-1 is associated with extended lifespan and health-span. Additionally, we confirmed that IGF-1 interacts with age to modify risk in a manner consistent with antagonistic pleiotropy: younger individuals with high IGF-1 are protected from dementia, vascular disease, diabetes, cancer, and osteoporosis, while older individuals do not exhibit IGF-1-associated protection from disease. These findings offer evidence for IGF-1 modulating health-span and lifespan in humans.

